# Improving the Maximum Transmission Distance of Self-Referenced Continuous-Variable Quantum Key Distribution Using a Noiseless Linear Amplifier

**DOI:** 10.3390/e20060461

**Published:** 2018-06-14

**Authors:** Yijun Wang, Xudong Wang, Duan Huang, Ying Guo

**Affiliations:** 1 School of Information Science and Engineering, Central South University, Changsha 410083, China; 2 School of IOT Engineering, Taihu University, Wuxi 214064, China

**Keywords:** continuous variable, quantum key distribution, noiseless linear amplifier (NLA)

## Abstract

We show that a noiseless linear amplifier (NLA) can be placed properly at the receiver’s end to improve the performance of self-referenced (SR) continuous variable quantum key distribution (CV-QKD) when the reference pulses are weak. In SR CV-QKD, the imperfections of the amplitude modulator limit the maximal amplitude of the reference pulses, while the performance of SR CV-QKD is positively related to the amplitude of the reference pulses. An NLA can compensate the impacts of large phase noise introduced by the weak reference pulses. Simulation results derived from collective attacks show that this scheme can improve the performance of SR CV-QKD with weak reference pulses, in terms of extending maximum transmission distance. An NLA with a gain of *g* can increase the maximum transmission distance by the equivalent of $20\log_{10}g$ dB of losses.

## 1. Introduction

Quantum key distribution (QKD) is the state-of-the-art application of quantum technologies, which is able to establish a secret key between two distant legal communicators, usually called Alice and Bob, through an insecure classical channel or quantum channel [1,2,3,4]. QKD has three major branches, the first is the discrete variable (DV) QKD based on manipulating and detecting the single photon state (polarization or phase), the second is the continuous variable (CV) QKD based on preparing and measuring coherent state or EPR state [5,6,7,8], and the last one is the differential phase reference (DPR) QKD [9,10,11]. With the depth of research in recent years, CV-QKD has fully demonstrated its major merits, such as high detection efficiency and low experimental cost. Most importantly, it can be implemented by using the existing commercial fibre communication networks, so it has attracted much attention and given many meaningful research results [12,13,14,15,16].

Generally, the most studied CV-QKD protocol is the GG02 protocol [5] and its unconditional security has been conducted in theory [17,18,19]. However, recent studies show that the imperfections in the Gaussian CV-QKD experimental system setups will cause a series of new severe security loopholes [20,21,22]. Furthermore, in Gaussian CV-QKD protocols, a high-brightness classical beam called local oscillator (LO) is co-transmitted with the weak quantum signal. The LO is indispensable because it can provide phase reference when Bob performs coherent detection on the received quantum signals. Some side-channel attacks aiming at LO have been confirmed , which can greatly reduce the overall security of the Gaussian CV-QKD protocol [23,24]. Fortunately, a novel scheme named self-referenced (SR) CV-QKD that could generate real “local” LO at Bob’s end has been proposed very recently [25,26,27] and shows its robustness in allusion to these attacks. However, due to the limited dynamic modulation range of the amplitude modulator, the amplitude of the reference pulses cannot be too large in practical. Besides, the imperfections in Alice’s modulator will create an extra excess noise proportional to the amplitude of reference pulses. This further limits the maximum amplitude of the reference pulses [28]. Since the secret key rate and the maximum transmission distance of SR CV-QKD scheme is positively correlated with the amplitude of the reference pulses, the weak reference pulses can degrade the performance of SR CV-QKD greatly.

Recently, some works have shown that a noiseless linear amplifier (NLA) [29,30,31,32,33,34,35,36] could be properly embedded in CV-QKD to fight against channel loss and improve maximum transmission distance [37,38,39,40,41]. In our paper, we consider the use of an NLA inserted before the detection stage in an SR CV-QKD scheme to improve the transmission distance when the reference pulses are weak. Usually, an NLA can amplify the amplitude of input coherent probabilistically while retaining the original level of channel noise [29]. This is very important for the SR CV-QKD because it is very sensitive to the phase noise. When we only take the successful runs of an NLA into account, it can compensate the adverse effects of high phase noise introduced by weak self-referenced pulse and attain a much longer transmission distance. Besides, the impact of the probability that the NLA successfully amplified the quantum signal may be inconspicuous because it is the gain of NLA *g* that influences the maximum transmission distance primarily rather than the success rate, which is always lower than $1/g^{2}$ [37].

This article is organized as follows. In Section 2, we review the SR CV-QKD scheme, and then we introduce the NLA SR CV-QKD schme. In Section 3, we analyze the secret key rate of our proposed scheme and demonstrate the maximum transmission distance improvement. Finally, we summarize our paper in Section 4.

## 2. The SR CV-QKD Scheme & Our Proposed Scheme

**Figure 1:**
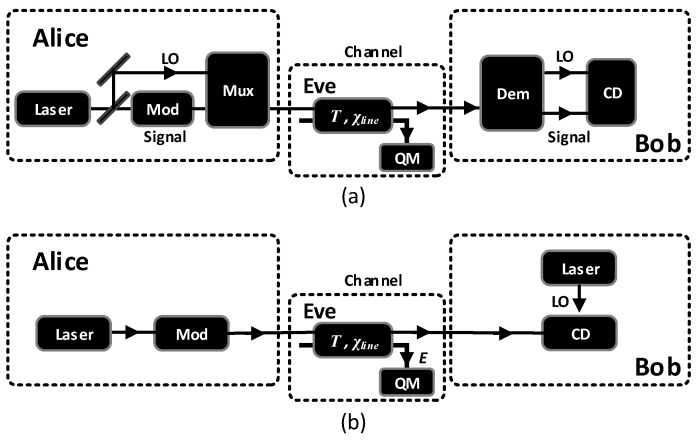
(**a**) Conventional one-way CV-QKD scheme. The quantum signal and LO are co-transmitted from Alice to Bob. During the QKD process, complicated multiplexing and demultiplexing techniques are employed. (**b**) SR CV-QKD scheme. Alice sends quantum signals and reference pulses to Bob in the same channel. Bob measures the received pulses in his own phase reference frame defined by the locally generated LO. Mod: Gaussian modulator; Mux: multiplexer; Dem: demultiplexer; CD: coherent detection; QM: quantum memory.

Figure 1a illustrates the steps of the conventional Gaussian CV-QKD scheme. The LO and modulated quantum signals are co-transmitted by adapting techniques like time-division multiplexing (TDM), wavelength-division multiplexing (WDM) and polarization encoding. After receiving the multiplexed signals, Bob uses a demultiplexer to split the LO and quantum signals. As mentioned above, the nature of LO would cause side-channel attacks and it is knotty to multiplex and demultiplex two kinds of signals that differ greatly in amplitude.

The SR CV-QKD scheme [25,26,27] described in Figure 1b has removed the demand of transporting LO successfully. In SR CV-QKD scheme, Alice sends a Gaussian modulated coherent state to Bob just like performed in conventional CV-QKD scheme at first, in the next time bin, she prepares another coherent state as the reference pulse and sends to Bob. The amplitude of the reference pulse, $E_{R}$, is few times larger than the variance of the quantum signal, $V_{A}$.

The reference pulse is used to estimate the deviation angle $\hat{\theta }$ between Alice and Bob’s reference frame. The $\hat{\theta }=\theta +\phi$, where θ is the actual deviation angle and ϕ is the measurement error mainly caused by the quantum uncertainty. We can easily deduce the value of $\hat{\theta }$ from some simple geometric calculations and find the correlations between the quadratures of sent quantum states and the quadratures of received quantum states.

Since the system performance of SR CV-QKD is positively related to the amplitude of the reference pulses $E_{R}$, the authors choose arbitrary large $E_{R}$ to attain a longer transmission distances and a higher secret key rate. However, due to the limited dynamic modulation range of the amplitude modulator (AM), the value of $E_{R}$ cannot be too large in practical terms. Besides, the imperfections existing in Alice’s AM will introduce an extra excess noise that can be approximated as [28]

(1) $$\varepsilon_{AM}=E_{max}^{2}10^{-d_{dB}/10},$$

where $E_{max}$ is the maximal amplitude to be modulated and the $d_{dB}$ represents the dynamic modulation range of the AM. Since the extra excess noise is proportional to the amplitude $E_{max}$, and $d_{dB}$ has a finite value, this imperfection further limits the amplitude of reference pulses. However, the weaker the reference pulse, the larger the measurement error for θ caused by the quantum uncertainty, and the greater the phase noise variance, ultimately resulting in degrading the performance of SR CV-QKD. In the case of transporting weak reference pulses ($E_{R}/V_{A}=20,V_{A}=40$), the maximum transmission distance of SR CV-QKD is less than 15 km [25]. Therefore, the range of applications of the original SR CV-QKD scheme may be limited.

**Figure 2:**
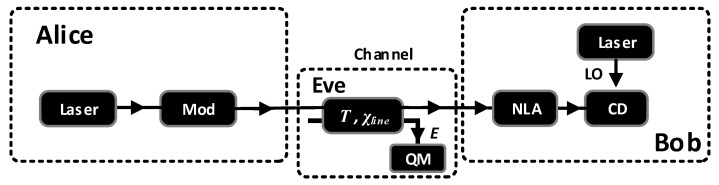
Schematic of the SR CV-QKD protocol with an NLA before detection. Mod: Gaussian modulator; CD: coherent detection; QM: quantum memory.

The NLA has been proven to be a useful tool to extend the maximum transmission distance of Gaussian CV-QKD [37,38,39]. In this paper, an NLA is placed at Bob’s end before the coherent detection described in Figure 2 to increase the maximum transmission distance of SR CV-QKD when reference pulses are weak. As usual, we will use the entangle-based (EB) version to describe and analyze our scheme and start with analysing the covariance matrix of the state $\rho_{AB}$ shared between Alice and Bob before any measurement. In a conventional CV-QKD scheme, the covariance matrix $\gamma_{AB}$ has the following form:

(2) $$\begin{array}{c} \begin{array}{c} \gamma_{AB}\left(\lambda ,T,\varepsilon \right)=\left(\begin{array}{cc} V(\lambda )I & \sqrt{T(V{\left(\lambda \right)}^{2}-1)}\sigma_{z}\\ \sqrt{T(V{\left(\lambda \right)}^{2}-1)}\sigma_{z} & T\left[V(\lambda )+B+\varepsilon \right]I \end{array}\right), \end{array} \end{array}$$

where $I=(\begin{array}{cc} 1 & 0\\ 0 & 1 \end{array})$ and $\sigma_{z}=\left(\begin{array}{cc} 1 & 0\\ 0 & -1 \end{array}\right)$, $V\left(\lambda \right)=\frac{1+\lambda^{2}}{1-\lambda^{2}}$ is the variance of the thermal state $Tr_{A}\left|\lambda \rangle \langle \lambda \right|$ related to the modulation variance and λ is the parameter of squeezed state, the $B=\frac{1-T}{T}$ refer to the noise introduced by the channel loss, the ε is the channel excess noise.

**Figure 3:**
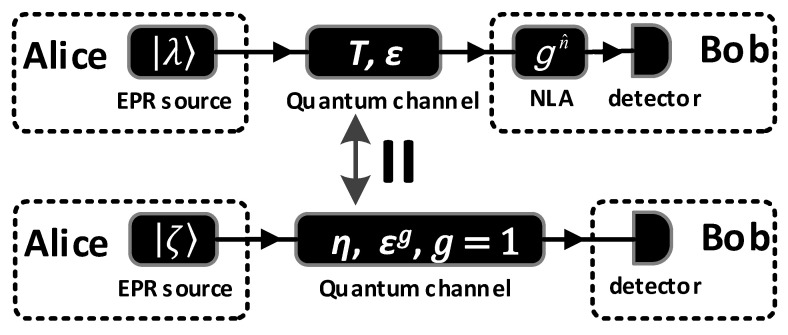
The CV-QKD scheme added an NLA with parameters (λ,T,ε,g) is equivalent to the scheme without using an NLA with parameters $\left(\zeta ,\eta ,\varepsilon^{g},g=1\right)$.

When an NLA is inserted into a conventional CV-QKD, only when the NLA works on its successful runs, the exchanged quantum signals will be used for extracting the secret key. Therefore, the scheme of CV-QKD added an NLA is very similar to those CV-QKD schemes with postselection [42]. Since the output of the NLA remains in the Gaussian regime, we can use an equivalent EPR scheme without NLA to analyse the impacts of an NLA on the original scheme. It is shown in Figure 3 that the covariance matrix $\gamma_{AB}\left(\lambda ,T,\varepsilon \right)$ is equivalent to the covariance matrix $\gamma_{e(AB)}\left(\zeta ,\eta ,\varepsilon^{g},g=1\right)$ (g=1 indicates no NLA). These equivalent parameters are given by [37]

(3) $$\begin{array}{c} \begin{array}{c} \zeta =\lambda \sqrt{\frac{\left(g^{2}-1\right)\left(\varepsilon -2\right)T-2}{(g^{2}-1)\varepsilon T-2}},\\ \eta =\frac{g^{2}T}{\left(g^{2}-1\right)T\left[\frac{1}{4}\left(g^{2}-1\right)\left(\varepsilon -2\right)\varepsilon T-\varepsilon +1\right]+1}\\ \varepsilon^{g}=\varepsilon -\frac{1}{2}\left(g^{2}-1\right)\left(\varepsilon -2\right)\varepsilon T. \end{array} \end{array}$$

It is clear that the parameters $\left(\zeta ,\eta ,\varepsilon^{g}\right)$ are equal to the parameters (λ,T,ε) respectively when g=1. These parameters must meet with the physical meaning limits of 0⩽ζ<1, 0⩽η<1 and $\varepsilon^{g}⩾0$, and the maximum value of the gain $g_{max}$ is given by [37]

(4) $$\begin{array}{c} g_{max}\left(T,\varepsilon \right)=\sqrt{\frac{\varepsilon \left[T\left(\varepsilon -4\right)+2\right]+4\sqrt{\frac{T(\varepsilon -2)+2}{\varepsilon }}-2\sqrt{\varepsilon [T(\varepsilon -2)+2]}+4T-4}{T{\left(\varepsilon -2\right)}^{2}}}. \end{array}$$

In the SR CV-QKD scheme, the removal of LO will not change the relevant parameters of the channel. Therefore, our proposed scheme can be regarded as an equivalent SR CV-QKD scheme without inserting an NLA, while the equivalent parameters are consistent with the parameters in Equation (3). When we take the phase-space rotations due to the reference frame misalignment into account, the density matrix of the state shared by Alice and Bob in the equivalent SR CV-QKD scheme without using NLA is [25]

(5) $$\begin{array}{c} {\bar{\rho }}_{e(AB)}=\bar{\rho_{e(AB)}\left(\hat{\theta },\theta \right)}=\int_{-\pi }^{\pi }d\phi P\left(\phi \right)\int_{-\pi }^{\pi }\frac{d\theta }{2\pi }\rho_{e(AB)}\left(\hat{\theta },\theta \right) \end{array}$$

with

(6) $$\rho_{e(AB)}\left(\hat{\theta },\theta \right)=\left[U_{A}\left(-\hat{\theta }\right)U_{B}\left(\theta \right)\right]\rho_{e(AB)}\left[U_{A}^{†}\left(-\hat{\theta }\right)U_{B}^{†}\left(\theta \right)\right],$$

where $U_{A(B)}$ represents the phase-space rotation operator, P(ϕ) is the probability distribution function of random variable ϕ. While the state ${\bar{\rho }}_{e(AB)}$ maintains Gaussian, the covariance matrix ${\bar{\gamma }}_{e(AB)}$ can be expressed as follows [25]

(7) $$\begin{array}{c} {\bar{\gamma }}_{e(AB)}=\bar{\gamma_{e(AB)}\left(\hat{\theta },\theta \right)}=\int_{-\pi }^{\pi }d\phi P\left(\phi \right)\int_{-\pi }^{\pi }\frac{d\theta }{2\pi }\gamma_{e(AB)}\left(\hat{\theta },\theta \right) \end{array}$$

with

(8) $$\gamma_{e(AB)}\left(\hat{\theta },\theta \right)=\left[U_{A}\left(-\hat{\theta }\right)⊕U_{B}\left(\theta \right)\right]\gamma_{e(AB)}\left[U_{A}^{⊤}\left(-\hat{\theta }\right)⊕U_{B}^{⊤}\left(\theta \right)\right],$$

the $U_{A(B)}$ is the symplectic representation of the rotation operator $U_{A(B)}$.

In our scheme, during each time of successful amplification, the NLA can be regarded as an operator $\hat{C}$. It can amplify a coherent state from |α〉 to $\hat{C}|\alpha \rangle =e^{\frac{{\left|\alpha \right|}^{2}}{2}\left(g^{2}-1\right)}|g\alpha \rangle$ probabilistically [37], where *g* is the gain of NLA. In other words, the NLA can increase the variance of the original quantum signals and improve the channel transmittance. This can offset the adverse effects of the large phase noise incurred by the weak reference pulses and extend the maximum transmission distance of SR CV-QKD. In the next section, we will use numerical simulations to illuminate this improvement in detail.

## 3. Performance Analysis

In SR CV-QKD, the modulation process for the quantum signal and reference pulse is time independent. When the AM only modulates the reference pulses, the large $E_{R}$ and the finite dynamic modulation range $d_{dB}$ would introduce a large $\varepsilon_{AM}$. So, we need to set a reasonable and realistic value for reference pulse amplitude to eliminate the effect of $\varepsilon_{AM}$ on its measurement result. When the AM only modulates the quantum signals, the extra excess noise $\varepsilon_{AM}$ in Equation (1) is independent of $E_{R}$, but is related to the amplitude of quantum signals. Considering that the intensity $E_{max}^{2}$ of modulated quantum signal is just few times larger than $V_{A}$ [43], the value of resulting excess $\varepsilon_{AM}$ is tiny ($\varepsilon_{AM}∼10^{-3}$ for $V_{A}=4$, $E_{max}^{2}=10V_{A}$, and $d_{dB}=40$). Compared to the noise introduced by the Gaussian channel (B+ε), the influence of $\varepsilon_{AM}$ introduced by the quantum signals on the system performance could be ignored.

The secret key rate of SR CV-QKD scheme under collective attacks with reverse reconciliation is [3,25,44]

(9) $$K=\beta I_{AB}-\chi_{BE},$$

where $I_{AB}$ is the mutual information between Alice’s and Bob’s measurements and it can be expressed as

(10) $$I_{AB}=\frac{1}{2}\log_{2}\left(\frac{V_{A}}{V_{A|B}}\right)=\frac{1}{2}\log_{2}\left(\frac{V+B+\varepsilon }{B+\varepsilon +1+(V-1)\xi }\right),$$

where $\xi =1-{\left(\bar{\cos\phi }\right)}^{2}$. Presuming that the distribution interval of P(ϕ) is symmetrical and very narrow, namely |ϕ|∼0, then we get $\xi \approx \bar{\phi^{2}}$. When the rate of pulse generation is much greater than the fluctuation frequency of phase difference θ, the value of θ can be treated as a specific constant. Therefore, the variance of estimated phase difference $\hat{\theta }$ is $V_{\hat{\theta }}=V_{\phi }=\bar{\phi^{2}}$. If P(ϕ) is rapidly monotonically decreasing in the interval $\left[0,|\phi {|}_{max}\right]$, the variance $V_{\hat{\theta }}$ can be regarded as a tight up bound of ξ, which means $\xi ≲V_{\hat{\theta }}$. The expression of $V_{\hat{\theta }}$ is as follows [25]

(11) $$V_{\hat{\theta }}=\frac{B+\varepsilon +1}{E_{R}}+\frac{1}{TE_{R}}.$$

So, the lower bound of $I_{AB}$ is

(12) $$I_{AB}≳\frac{1}{2}\log_{2}\left(\frac{V+B+\varepsilon }{B+\varepsilon +1+\left(V-1\right)V_{\hat{\theta }}}\right).$$

The $\chi_{BE}$ is the Holevo bound denotes for Eve’s maximum accessible information, which can be derived from the following formula:

(13) $$\chi_{BE}=G\left(\frac{\lambda_{1}-1}{2}\right)+G\left(\frac{\lambda_{2}-1}{2}\right)-G\left(\frac{\lambda_{3}-1}{2}\right),$$

where $G\left(x\right)=\left(x+1\right)\log_{2}\left(x+1\right)-x\log_{2}\left(x\right)$ is the Von Neumann entropy of a thermal state. The eigenvalues $\lambda_{1}$ and $\lambda_{2}$ are obtained from

(14) $$\begin{array}{c} \lambda_{1,2}^{2}=\frac{1}{2}\left(\Delta \pm \sqrt{\Delta^{2}-4D^{2}}\right), \end{array}$$

where we have used the notations

(15) $$\begin{array}{c} \begin{array}{c} \Delta =V^{2}+T^{2}{\left(V+B+\varepsilon \right)}^{2}+2T\left(V^{2}-1\right)\left(\xi -1\right),\\ D=T[V\left(B+\varepsilon \right)+1+\left(V^{2}-1\right)\xi ]. \end{array} \end{array}$$

The square of symplectic eigenvalue $\lambda_{3}$ reads

(16) $$\begin{array}{c} \lambda_{3}^{2}=V\frac{V\left(B+\varepsilon \right)+1+\left(V^{2}-1\right)\xi }{V+B+\varepsilon }. \end{array}$$

We can notice that the $\chi_{BE}$ is monotonically increasing with increasing ξ. When we replace the ξ in Equations (15) and (16) with $V_{\hat{\theta }}$, the lower bound of *K* is acquired.

As mentioned earlier, our scheme of SR CV-QKD with an NLA has parameters (λ,T,ε,g) and can be treated as one-way SR CV-QKD without NLA having parameters $\left(\zeta ,\eta ,\varepsilon^{g},g=1\right)$. When our scheme works on the successful runs, the secret information in Equation (9) could be acquired by adopting the equivalent parameters

(17) $$\begin{array}{c} \Delta K_{SR}^{g}\left(\lambda ,T,\varepsilon ,\beta \right)=\Delta K_{SR}\left(\zeta ,\eta ,\varepsilon^{g},\beta \right). \end{array}$$

Since the NLA could retain the original level of channel noise, the random variables θ and ϕ will not be affected, so that we could use the method stated in [25] to handle them. Finally, we can use the covariance matrix ${\bar{\gamma }}_{e(AB)}$ to figure out $\Delta K_{SR}\left(\zeta ,\eta ,\varepsilon^{g},\beta \right)$.

Before starting the simulation, we need to take the successful amplification probability $P_{SS}$ of the NLA into account. The actual value of $P_{SS}$ is related to the experimental setups and it is not important to our study because on the one hand we mainly focus on the maximum distance, and on the other hand, it is only a proportional coefficient and cannot transform a negative secret key into a positive one. Besides, The $P_{SS}$ could be treated as constant if the NLA has sufficient dynamics to neglect distortions [37]. Then we can get the secret key rate with NLA by multiplying $\Delta K_{SR}$ by the $P_{SS}$

(18) $$\begin{array}{c} \Delta K_{NLA}=P_{SS}\Delta K_{SR}\left(\zeta ,\eta ,\varepsilon^{g},\beta \right), \end{array}$$

the $P_{SS}$ for an NLA with a gain of *g* is upper bounded to $1/g^{2}$, which will be used in the later analysis.

**Figure 4:**
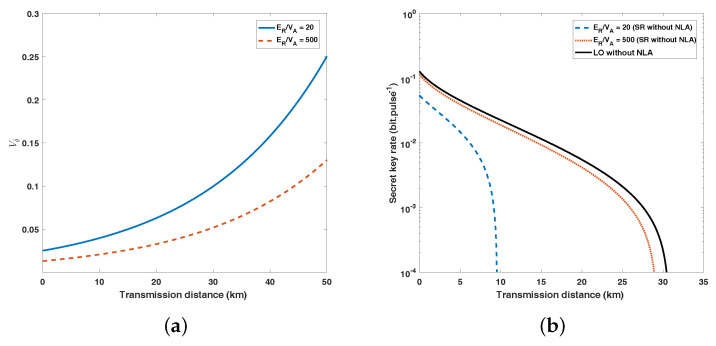
(Color online) (**a**) The variance of estimated phase difference $\hat{\theta }$ as a function of transmission distance at different values of $E_{R}$. (**b**) Secret key rate of SR CV-QKD scheme when $E_{R}$ takes ideal value ($E_{R}=500V_{A}$) and reasonable value ($E_{R}=20V_{A}$). The parameters involved above: $V_{A}=4$, ε=0.01 (all in shot-noise units), β=0.95.

As mentioned above, the amplitude of the reference pulses $E_{R}$ is critical to the performance of the SR CV-QKD scheme. The larger the value of $E_{R}$, the smaller the variance of the measurement error for θ and the higher the secret key rate. Figure 4a shows the relationship between variance $V_{\hat{\theta }}$ and transmission distance and the secret key rate at different values of $E_{R}$. In contrast to an ideal value like $500V_{A}$, when $E_{R}$ is given a more reasonable value such as $20V_{A}$, the value of $V_{\hat{\theta }}$ is approximately doubled. Figure 4b illustrates the secret key rate and the maximum transmission distance of SR CV-QKD when $E_{R}$ takes ideal and reasonable values, respectively. When the reference pulses are weak $\left(E_{R}/V_{A}=20\right)$, the maximum transmission distance is limited to about 10 km, which is less than half of the transmission distance in the LO scheme . At this point, the SR CV-QKD scheme is only suitable for short-range communications and cannot even be used in urban communication networks.

**Figure 5:**
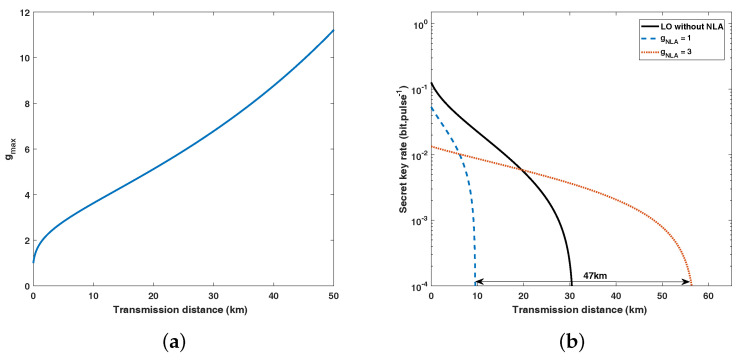
(Color online) (**a**) Maximum value of the gain as a function of transmission distance where excess noise ε=0.01. (**b**) Maximum transmission distance of the SR CV-QKD scheme using an NLA with different gain (g = 1, 2, 3). Other parameters involved above: V=4, ε=0.01(all in shot-noise units), β=0.95, $E_{R}/V_{A}=20$.

Another important point in our scheme is the gain of NLA. The value of $g_{max}$ in Equation (4) only depends on the channel parameters (T,ε). In Figure 5a, we display the correlation between the $g_{max}$ and transmission distance, while the excess noise ε is 0.01. The simulations of $\Delta K_{NLA}$ with the same channel parameters are shown Figure 5b. In this figure, we set the value of *g* to 3, which is lower than the $g_{max}$ in Figure 5a. We can see that the NLA with a gain of g can help increase the distance by the equivalent $20\log_{10}g$ dB of losses ($100\log_{10}g$ km when the fibre attenuation coefficient α=0.2 dB/km).

**Figure 6:**
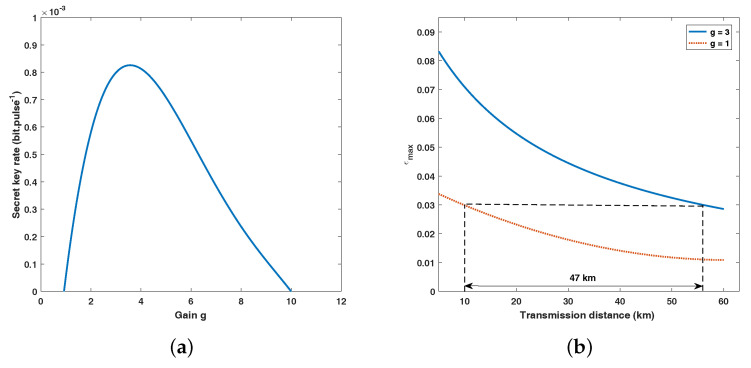
(Color online) (**a**) Maximized secret key rate as the function of the gain g when the transmission distance is 50 km. (**b**) Maximized excess noise as a function of transmission distance while the secret key rate remains positive. Other parameters involved in the above two figures: $V_{A}=4$, ε=0.01 (all in shot-noise units), β=0.95, $E_{R}/V_{A}=20$.

However, increasing the value of *g* will increase the transmission distance intuitively but the secret key rate may become negative. There are two reasons for this, one is the possibility $P_{SS}$, and the other is the excess noise $\varepsilon^{g}$ will increase as well. In Figure 6a, we can see that when the transmission distance is determined, the secret key rate will increase as the gain increases to a certain value. As the gain continues to increase, the secret key rate drops rapidly and eventually becomes negative. In addition, the maximum tolerable excess noise of SR CV-QKD against transmission distance with different gain *g* is shown in Figure 6b. It has clearly demonstrated that our scheme can tolerate a much higher excess noise while the secret key rate remains positive.

We should note that the simulation results displayed above may not correspond to the results of the practical experiment, because the possibility of $P_{SS}$ being a tunable parameter depends on the facilities configuration. However, they have clearly illustrated the effects of an NLA on the maximum transmission distance and secret key rate.

## 4. Conclusions

In SR CV-QKD, when the reference pulses are weak, the large phase noise shows a conspicuous adverse effect on its performance, which means a shorter transmission distance and a lower key rate. We show that an NLA can help to improve the performance of SR CV-QKD with weak reference pulses. The NLA can compensate the impacts of extra phase noise by enhancing the original quantum signals and increasing the channel transmittance. Our proposed scheme demonstrates that the NLA can help increase the distance by the equivalent $20\log_{10}g$ dB of losses (g=3 for 47 km) and the secret key rate is still acceptable. However, it should be mentioned here that we have only conducted theoretical analysis; the gap between practical implementations and theoretical models should also be considered. Any imperfection that exists in the actual experiment would introduce more complex parameters. This issue is not within the scope of our current consideration, and deserves further investigation.

## References

[1] Bennett C.H., Brassard G. Quantum cryptography: Public key distribution and coin tossing. Proceedings of the IEEE International Conference on Computers, Systems and Signal Processing.

[2] Gisin N., Ribordy G., Tittel W., Zbinden H. (2002). Quantum cryptography. Rev. Mod. Phys..

[3] Scarani V., Bechmann-Pasquinucci H., Cerf N.J., Dušek M., Lütkenhaus N., Peev M. (2009). The security of practical quantum key distribution. Rev. Mod. Phys..

[4] Lo H.-K., Curty M., Tamaki K. (2014). Secure quantum key distribution. Nat. Photonics.

[5] Grosshans F., Grangier P. (2002). Continuous variable quantum cryptography using coherent states. Phys. Rev. Lett..

[6] Braunstein S.L., van Loock P. (2005). Quantum information with continuous variables. Rev. Mod. Phys..

[7] Wang X.B., Hiroshima T., Tomita A., Hayashi M. (2007). Quantum information with gaussian states. Phys. Rep..

[8] Weedbrook C., Pirandola S., García-Patrón R., Cerf N.J., Ralph T.C., Shapiro J.H., Lloyd S. (2012). Gaussian quantum information. Rev. Mod. Phys..

[9] Inoue K., Waks E., Yamamoto Y. (2003). Differential-phase-shift quantum key distribution using coherent light. Phys. Rev. A.

[10] Stucki D., Brunner N., Gisin N., Scarani V., Zbinden H. (2005). Fast and simple one-way quantum key distribution. Appl. Phys. Lett..

[11] Bacco D., Christensen J.B., Castaneda M.A.U., Ding Y. (2016). Two-dimensional distributed-phase-reference protocol for quantum key distribution. Sci. Rep..

[12] Ma H.X., Bao W.S., Li H.W. (2016). Quantum hacking of two-way continuous-variable quantum key distribution using trojan-horse attack. Chin. Phys. B.

[13] Guo Y., Liao Q., Huang D., Zeng G.H. (2017). Quantum relay schemes for continuous-variable quantum key distribution. Phys. Rev. A.

[14] Guo Y., Xie C.L., Liao Q., Zhao W., Zeng G.H., Huang D. (2017). Entanglement-distillation attack on continuous-variable quantum key distribution in a turbulent atmospheric channel. Phys. Rev. A.

[15] Liu W.Q., Peng J.Y., Huang P., Huang D., Zeng G.H. (2017). Monitoring of continuous-variable quantum key distribution system in real environment. Opt. Express.

[16] Huang P., Huang J.Z., Wang T., Li H.S., Huang D., Zeng G.H. (2017). Robust continuous-variable quantum key distribution against practical sttacks. Phys. Rev. A.

[17] Grosshans F. (2005). Collective attacks and unconditional security in continuous variable quantum key distribution. Phys. Rev. Lett..

[18] Navascues M., Acín A. (2005). Security bounds for continuous variables quantum key distribution. Phys. Rev. Lett..

[19] Leverrier A. (2015). Composable security proof for continuous-Variable quantum key distribution with coherent States. Phys. Rev. Lett..

[20] Huang J.Z., Weedbrook C., Yin Z.Q., Wang S., Li H.W., Chen W., Guo G.C., Han Z.F. (2013). Quantum hacking of a continuous-variable quantum-key-distribution system using a wavelength attack. Phys. Rev. A.

[21] Ma X.C., Sun S.H., Jiang M.S., Liang L.M. (2013). Wavelength attack on practical continuous-variable quantum-key-distribution system with a heterodyne protocol. Phys. Rev. A.

[22] Qin H., Kumar R., Alléaume R. (2013). Saturation attack on continuous-variable quantum key distribution system. Proc. SPIE.

[23] Jouguet P., Kunz-Jacques S., Diamanti E. (2013). Preventing calibration attacks on the local oscillator in continuous-variable quantum key distribution. Phys. Rev. A.

[24] Ma X.C., Sun S.H., Jiang M.S., Liang L.M. (2013). Local oscillator fluctuation opens a loophole for Eve in practical continuous-variable quantum-key-distribution systems. Phys. Rev. A.

[25] Soh D.B.S., Brif C., Coles P.J., Lütkenhaus N., Camacho R.M., Urayama J., Sarovar M. (2015). Self-Referenced continuous-variable quantum key distribution protocol. Phys. Rev. X.

[26] Qi B., Lougovski P., Pooser R., Grice W., Bobrek M. (2015). Generating the local oscillator locally in continuous-variable quantum key distribution based on coherent detection. Phys. Rev. X.

[27] Huang D., Huang P., Lin D.K., Wang C., Zeng G.-H. (2015). High-speed continuous-variable quantum key distribution without sending a local oscillator. Opt. Lett..

[28] Marie A., Alléaume R. (2017). Self-coherent phase reference sharing for continuous-variable quantum key distribution. Phys. Rev. A.

[29] Ralph T.C., Lund A.P. (2009). Nondeterministic noiseless linear amplification of quantum systems. AIP Conf. Proc..

[30] Ferreyrol F., Barbieri M., Blandino R., Fossier S., Tualle-Brouri R., Grangier P. (2010). Implementation of a nondeterministic optical noiseless amplifier. Phys. Rev. Lett..

[31] Xiang G.Y., Ralph T.C., Lund A.P., Walk N., Pryde G.J. (2010). Heralded noiseless linear amplification and distillation of entanglement. Nat. Photonics.

[32] Usuga M.A., Müller C.R., Wittmann C., Marek P., Filip R., Marquardt C., Leuchs G., Andersen U.L. (2010). Heralded noiseless linear amplification and distillation of entanglement. Nat. Phys..

[33] Ferreyrol F., Blandino R., Barbieri M., Tualle-Brouri R., Grangier P. (2011). Experimental realization of a nondeterministic optical noiseless amplifier. Phys. Rev. A.

[34] Barbieri M., Ferreyrol F., Blandino R., Tualle-Brouri R., Grangier P. (2011). Nondeterministic noiseless amplification of optical signals: a review of recent experiments. Laser Phys. Lett..

[35] Zavatta A., Fiurasek J., Bellini M. (2011). A high-fidelity noiseless amplifier for quantum light states. Nat. Photonics.

[36] McMahon N.A., Lund A.P., Ralph T.C. (2014). Optimal architecture for a nondeterministic noiseless linear amplifier. Phys. Rev. A.

[37] Blandino R., Leverrier A., Barbieri M., Etesse J., Grangier P., Tualle-Brouri R. (2012). Improving the maximum transmission distance of continuous-variable quantum key distribution using a noiseless amplifier. Phys. Rev. A.

[38] Jaromír F., Nicolas C.J. (2012). Gaussian postselection and virtual noiseless amplification in continuous-variable quantum key distribution. Phys. Rev. A.

[39] Xu B.J., Tang C.M., Chen H., Zhang W.Z., Zhu F.C. (2013). Improving the maximum transmission distance of four-state continuous-variable quantum keydistribution by using a noiseless linear amplifier. Phys. Rev. A.

[40] Yang F.L., Shi R.H., Guo Y., Shi J.J., Zeng G.H. (2015). Continuous-variable quantum key distribution under the local oscillator intensity attack with noiseless linear amplifier. Quantum Inf. Process..

[41] Bai D.Y., Huang P., Ma H.X., Wang T., Zeng G.H. (2017). Performance improvement of plug-and-play dual-phase-modulated quantum key distribution by using a noiseless amplifier. Entropy.

[42] Walk N., Ralph T.C., Symul T., Lam P.K. (2013). Security of continuous-variable quantum cryptography with gaussian postselection. Phys. Rev. A.

[43] Fossier S. (2009). Mise en Oeuvre et Évaluation de Dispositifs de Cryptographie Quantique à Longueur D’onde Télécom. Ph.D. Thesis.

[44] García-Patrón R. (2007). Quantum Information with Optical Continuous Variables: From Bell Tests to Key Distribution. Ph.D. Thesis.

